# Supplementary material for: Drying in the low-latitude Atlantic Ocean contributed to terrestrial water storage depletion across Eurasia
Source: Nat Commun. 2022 Apr 6;13:1849. doi: 10.1038/s41467-022-29544-6 (PMC8986788; doi:10.1038/s41467-022-29544-6)
Supplement: Supplementary file 1 — Supplementary Information [file 41467_2022_29544_MOESM1_ESM.pdf]

Supplementary Information for

Drying in the low-latitude Atlantic Ocean contributed to terrestrial water storage depletion across Eurasia

The supplementary information includes:

Supplementary Notes 1-3

Supplementary Figures 1-26

Supplementary Tables 1-2

Supplementary Note 1 Maximum covariance analysis between PME over NATO and TWS across Eurasia

The explained variances of the leading modes 1-4 are 28.72%, 13.09%, 9.29% and 7.12%, respectively (Supplementary Fig. 14). The temporal sequences of PME and TWS in the leading modes 1-4 are highly related with correlation coefficients ranging between 0.86-0.94 ($p < 0.05$). These results corroborated synchronous fluctuations between PME and TWS (Supplementary Figs.14a, d, g, j). Especially, in the first leading mode, the variations in PME over the low-latitude NATO were in good agreement with variations in TWS across the mid-latitude Eurasia (Supplementary Figs.14a-c). The variations in PME over the high-latitude NATO were in good agreement with variations in TWS in the southern, eastern and northern Europe and most areas of western Russia (Supplementary Figs.14a-c). This spatial pattern agrees with the propagation routes of the landfalling impacts of the PMEs deficit over northern NATO from ocean to land during 2003-2017 as shown in Figure 3b of the main text (Supplementary Figs.14a-c).

Supplementary Note 2 Performance evaluation of CMIP6 in simulating P-E over the NATO

Given the uncertainties in the CMIP6 models in simulating the P-E over the lower latitude portion of the North Atlantic Ocean, we trained and tested the models for the projection of TWS using the P-E based on the historical ERA5 dataset, and projected the future variation in TWS based on the original P-Es of CMIP6 models. To confirm reliability of the projection results, we evaluated modelling performance of CMIP6 models in simulating P-E over the lower latitude portion of the North Atlantic Ocean, and then performed projection of TWS with future P-E based on weighted CMIP6 ensemble.

We firstly evaluated modelling performance of CMIP6 models in simulating P-E over the lower latitude of the North Atlantic Ocean in comparison with the ERA5-PME during 2003-2014 period. Supplementary Figures 22-23 illustrated divergent relationships between ERA5-PME and CMIP6-PMEs from model to model. For the simulation of PME in NATO1, the absolute value of the correlation coefficients between ERA5-PME and CMIP6-PMEs ranged from 0.05 to 0.49. Except for the PMEs simulated by IPSL-CM6A-LR and MRI-ESM2.0, the rest of the simulated CMIP6-PMEs over NATO1 were all significantly correlated with ERA5-PME with the $p < 0.05$ (Supplementary Fig. 22). For the simulation of the PME in NATO3, the absolute value of the correlation coefficients between ERA5-PME and CMIP6-PMEs ranged from 0.01 to 0.16 (Supplementary Fig. 23). Except for PMEs simulated by IPSL-CM6A-LR and MRI-ESM2.0, the rest of the simulated CMIP6-PMEs over NATO3 were all

significantly correlated with the ERA5-PME with $p < 0.05$ (Supplementary Figs. 23). Despite the low correlation coefficients between the CMIP6-PME and ERA-PME over the NATO3, the simulated PME by some of the CMIP6 models could accurately simulate the maximum and minimum values during 2003-2014 (Supplementary Fig. 23).

We developed initial linear regression models to quantify relations between ERA5-PME and CMIP6-PME during 2003-2017 and determine the weights for the calculation of multiple CMIP6 model ensemble (Eq. 1). Since the historical time periods for the CMIP6 models end in 2014, historical-SSP245 and historical-SSP585 during 2003-2017 are selected to determine the initial weights and significance level for the calculation of CMIP6 ensemble.

$$PME_{ERA5} = \sum_{i=1}^{8} a_i \times PME_i \quad \text{(Eq. 1)}$$

where PME_{ERA5} refers to the ERA5-PME over the NATO and PME_i refers to the simulated PME by the CMIP6 model i. a_i refers to the linear regression coefficients for the PME_i. And the CMIP6 models include ACCESS-ESM1.5, BCC-CSM2-MR, CanESM5, GFDL-ESM4, IPSL-CM6A-LR, MIROC6, MRI-ESM2.0 and NorESM2-LM.

Based on comparison of linear regression coefficients and significance levels among the simulated PMEs by CMIP6 models, PMEs simulated by ACCESS-ESM1.5, BCC-CSM2-MR and CanESM5 under historical-SSP245 scenario and PMEs simulated by ACCESS-ESM1.5, CanESM5 and MIROC6 under historical-SSP585 scenario were

further selected to determine the weights for the calculation of the PME based on the CMIP6 ensemble over NATO1 (Eqs. 2-3). PMEs simulated by ACCESS-ESM1.5, IPSL-CM6A-LR and NorESM2-LM over NATO3 under historical-SSP245 scenario and PMEs simulated by BCC-CSM2-MR, CanESM5 and MIROC6 under historical-SSP585 scenario were further selected to determine the weights for the calculation of the PME based on the multiple CMIP6 over the NATO3 (Eqs. 4-5).

$$PME_{ERA5} = \sum_{i=1,2,3} a_i \times PME_i \ (over\ NATO1\ under\ Historical - SSP245) \qquad \text{(Eq. 2)}$$

$$PME_{ERA5} = \sum_{i=1,3,6} a_i \times PME_i \ (over\ NATO1\ under\ Historical - SSP585) \qquad \text{(Eq. 3)}$$

$$PME_{ERA5} = \sum_{i=1,5,8} a_i \times PME_i \ (over\ NATO3\ under\ Historical - SSP245) \qquad \text{(Eq. 4)}$$

$$PME_{ERA5} = \sum_{i=2,3,6} a_i \times PME_i \ (over\ NATO3\ under\ Historical - SSP585) \qquad \text{(Eq. 5)}$$

According to Supplementary Figures 24-25, simulated PMEs by the weighted CMIP6 model ensemble over NATO1 and NATO3 were all significantly related to the ERA5-PMEs under the historical-SSP245 and SSP585 scenarios during 2003-2016. The correlation coefficients between ERA5-PME and simulated CMIP6-PME over NATO1 has been improved from 0.05-0.49 to 0.4-0.6. Similarly, the correlation coefficients

between ERA5-PME and simulated CMIP6-PME over NATO3 has been improved from 0.01-0.16 to 0.44-0.56 (Supplementary Figs. 24-25).

We further confirmed the reliability of the projected TWS under SSP245 and SSP585 scenarios by calculating the PMEs over NATO1 and NATO3 based on the weighted CMIP6 model ensemble (Supplementary Fig.26). We further projected the bias-corrected TWS using the trained model based on the historical TWS and ERA5-PME (Supplementary Fig.17).

Supplementary Note 3 Attribution analysis of TWS variations across mid-latitude Eurasia considering interactions between total freshwater withdrawals and PME over NATO

Here, the TFW was defined as the total freshwater withdrawals by domestic, agricultural and industrial activities. Only 5 out of the regions with decreasing TWSs were featured by TFW-dominated TWS such as Bosnia and Herzegovina (BosHerz for abbreviation in the figures), Lebanon, Pakistan, Tunisia and Xinjiang (China) (Supplementary Figs. 18e). Despite of the differences in variations of TFW, the decrease in TWS in these regions was in line with the decrease PME over NATO3 (Supplementary Figs. 18g-k). It should be emphasized that the variations in TWS in Bosnia and Herzegovina, Tunisia and Xinjiang (China) are highly sensitive to abrupt changes in PME over NATO3 in 2015 (Supplementary Figs. 18g-k). Meanwhile, when compared to regions with increasing TWS, the regions with decreasing TWS mainly distributed along the eastward propagation route of the landfalling PME-deficit originated from the low-latitude NATO. The temporal variation and spatial pattern both demonstrated that the PME deficit originated from the low-latitude NATO induced widespread decrease in TWS across the mid-latitude Eurasia. For the sake of drought mitigation in abovementioned 5 regions, TFW can exacerbate TWS deficit in the mid-latitude Eurasia.

France, Netherlands, Spain and Sweden were mere 4 out of the regions with increasing TWSs which were featured by TFW-dominated TWS (Supplementary Figs. 18f). However, the declines in TFW in France, Netherlands and Spain did not significantly

restore TWS during 2003-2009 and the increase in TFW in Sweden did not significantly lead to a decline in TWS during 2003-2009 (Supplementary Figs. 18l-o). Despite of the spatial heterogeneity of TFW over regions with increasing TWSs, TWS over above-mentioned 4 regions was in good agreement with the variation in PME over NATO4 during 2003-2015. Besides, different from the regions with declining TWS, the regions featured by an increasing TWS were distributed mostly in the western, southern and northern Europe, which are spatially close to NATO4 (Supplementary Figs. 18f). In summary, it demonstrated that the increase in PME over the NATO4 directly triggered increasing trends in TWS while decline in TFWs did not significantly impact the TWS over the western, southern and northern Europe during 2003-2016.

With the exception of human impacts on water storage, it is interesting to find an influence of variation in water storage on freshwater withdrawal behavior at the regional scale, which is reflected by the positive coefficient a, implying synchronous changes between TFW and TWS (Supplementary Fig. 19). It is in contrary to the assumption that TFW generally has negative impacts on TWS. Meanwhile, we evaluated relations between TWS and TFW and found that the correlation coefficient is > 0.3 over 65% of these regions (Supplementary Fig. 19), such as Belgium, Denmark, Greece, Poland, Israel, Slovenia, Georgia, Iraq, Kyrgyzstan, Kazakhstan, Moldova, Montenegro, Syria, Tajikistan and Ukraine. Moreover, variations in TWS were in agreement with variations in TFW and the increasing rates of GDP (gross domestic production) (Supplementary Figs. 19c-q). It is not meaningful to examine these

phenomena from the perspective of the negative impacts of local economic development and TFW on TWS. Instead, these phenomena demonstrated that variation in TWS could impact freshwater withdrawal behavior at the regional scale, and further influence local economic development due to constraint on freshwater withdrawals.

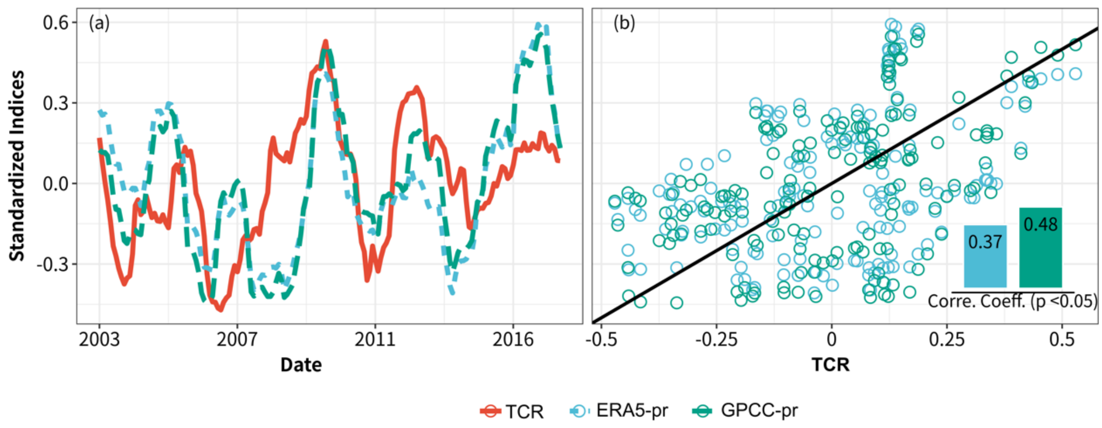

Supplementary Figure 1 Validation for the normalized trend item of the total contribution rates (TCR) of water vapor from all vapor source regions to Xinjiang with the normalized ERA5-based precipitation (ERA5-pr) and the GPCC-based precipitation (GPCC-pr) as benchmarks. (a) shows the temporal covariations among above indices, and (b) presents Pearson's correlation analysis between TCR and ERA5-pr and GPCC-pr, respectively. Since the input vapor formed the precipitation in Xinjiang, the process resulted in the time lag between variations in TCR and precipitation. Even though the correlation coefficients were 0.37 and 0.48, it should be noted that the p.value < 0.05 in the correlation analysis.

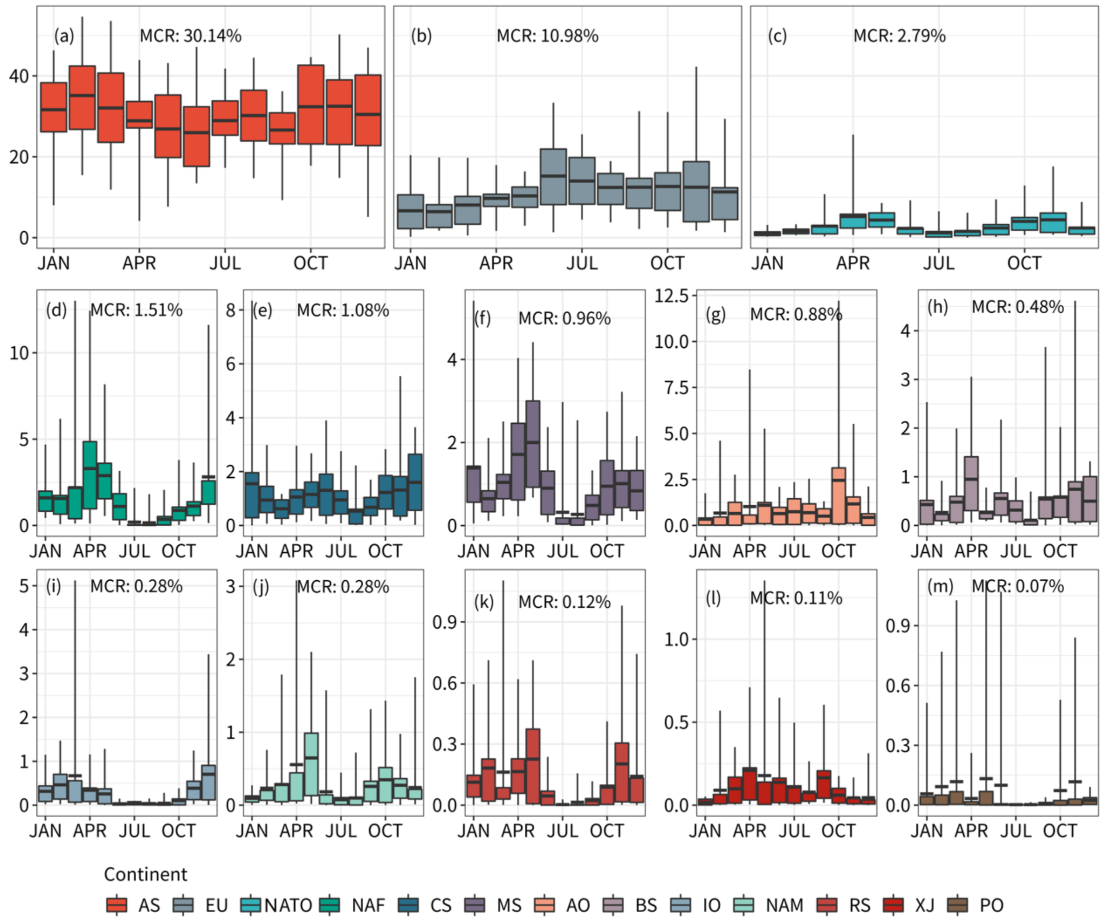

Supplementary Figure 2 Monthly variations of moisture contribution rates from all vapor source regions to Xinjiang during 2003-2017. (a-m) refer to monthly-averaged contribution rates (MCRs) from Asia (AS), Europe (EU), North Atlantic Ocean (NATO), North Africa (NAF), the Caspian Sea (CS), the Mediterranean Sea (MS), the Arctic Ocean (AO), the Black Sea (BS), the Indian Ocean (IO), North America (NAM), the Red Sea (RS), Xinjiang (XJ), and the Pacific Ocean (PO), respectively.

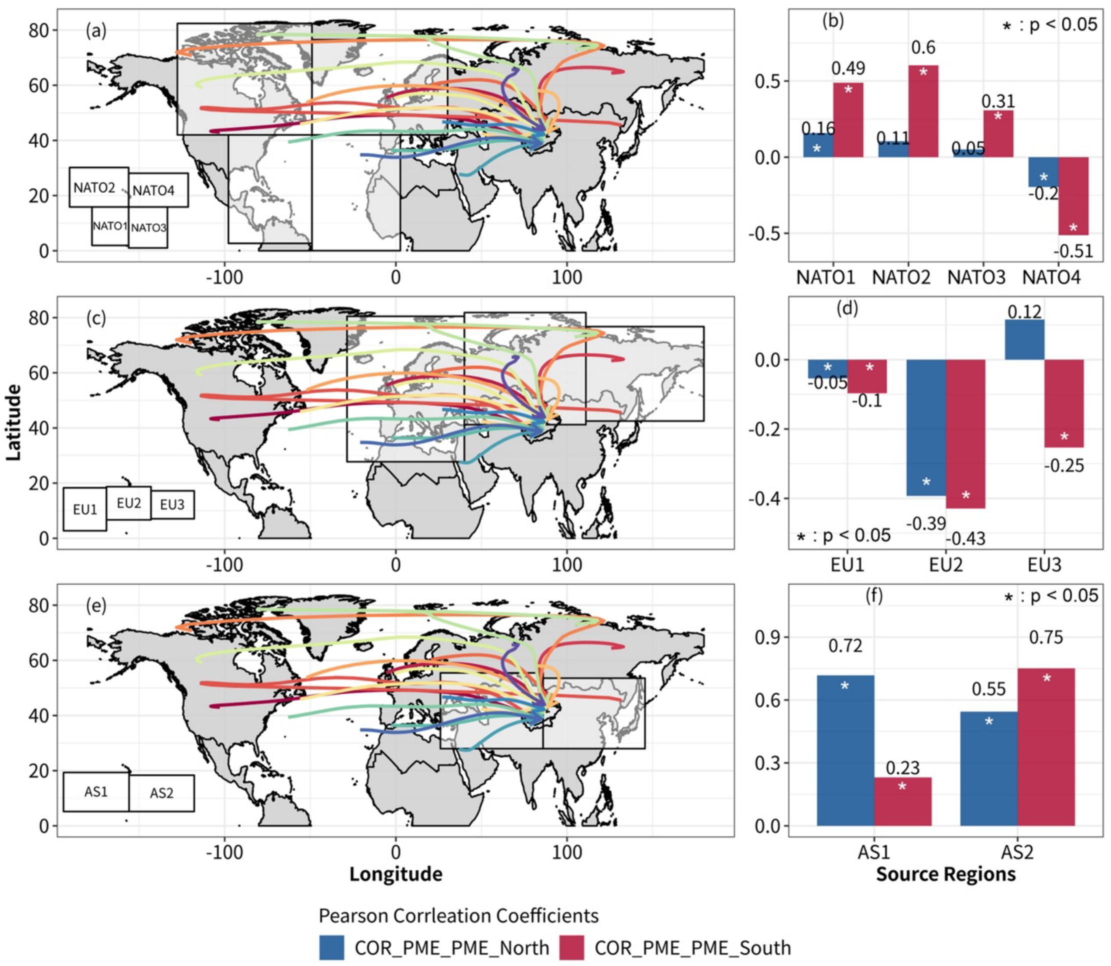

Supplementary Figure 3 Pearson correlation analysis between normalized trend items of the zonal sums of the precipitation minus evapotranspiration (PME for abbreviation) in north (south) Xinjiang and normalized trend items of the sums of regional PME in sub-regions of the northern Atlantic Ocean (NATO1-4, a-b), Europe (EU1-3, c-d), and Asia (AS1-2, e-f), respectively.

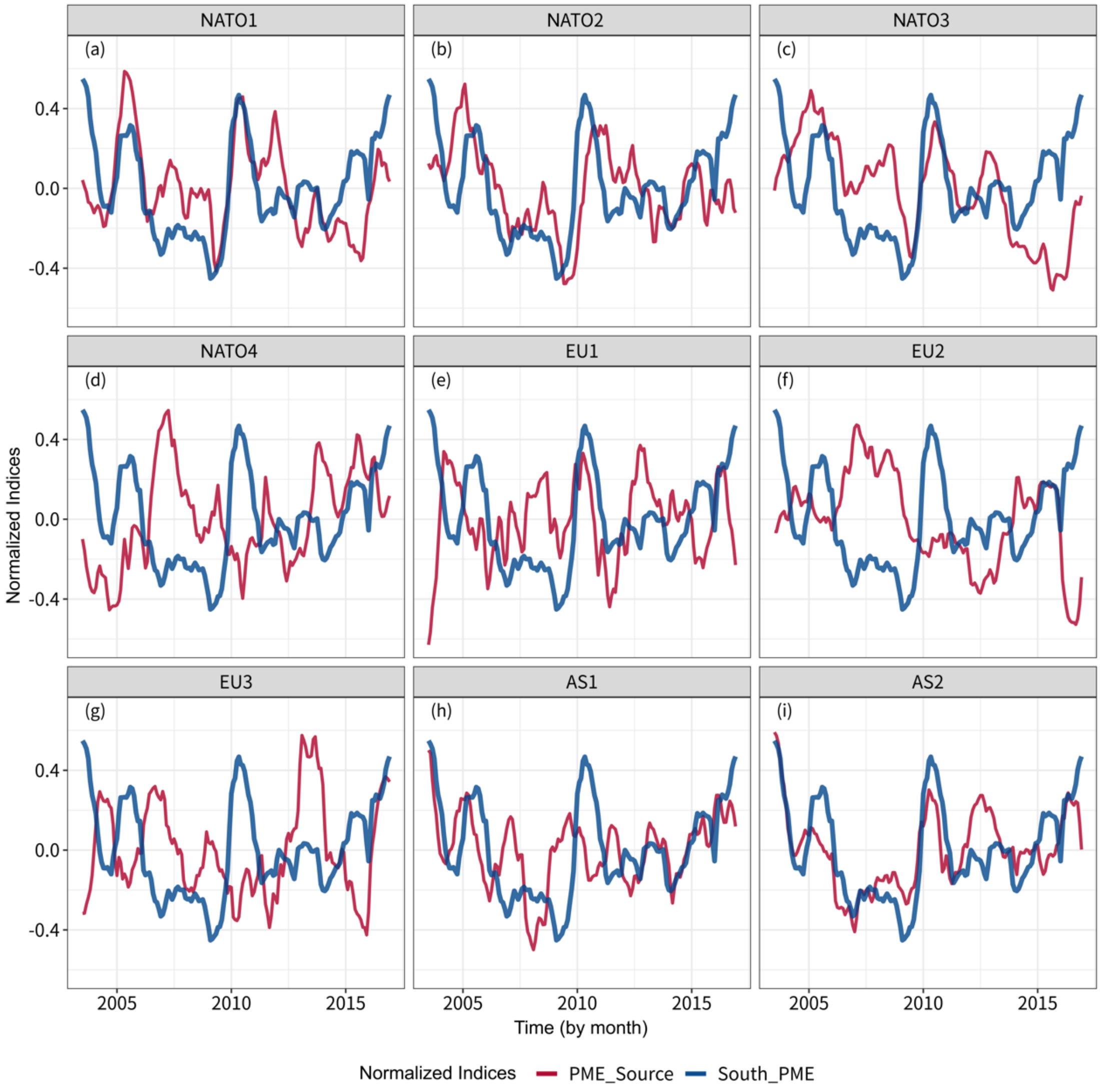

Supplementary Figure 4 Temporal variations in normalized trend items of the zonal sums of precipitation-minus-evapotranspiration (PME for abbreviation) in southern Xinjiang and normalized trend items of the zonal sums of PMEs in sub-regions of the northern Atlantic Ocean (NATO1-4, a-d), Europe (EU1-3, e-g), and Asia (AS1-2, h-i), respectively.

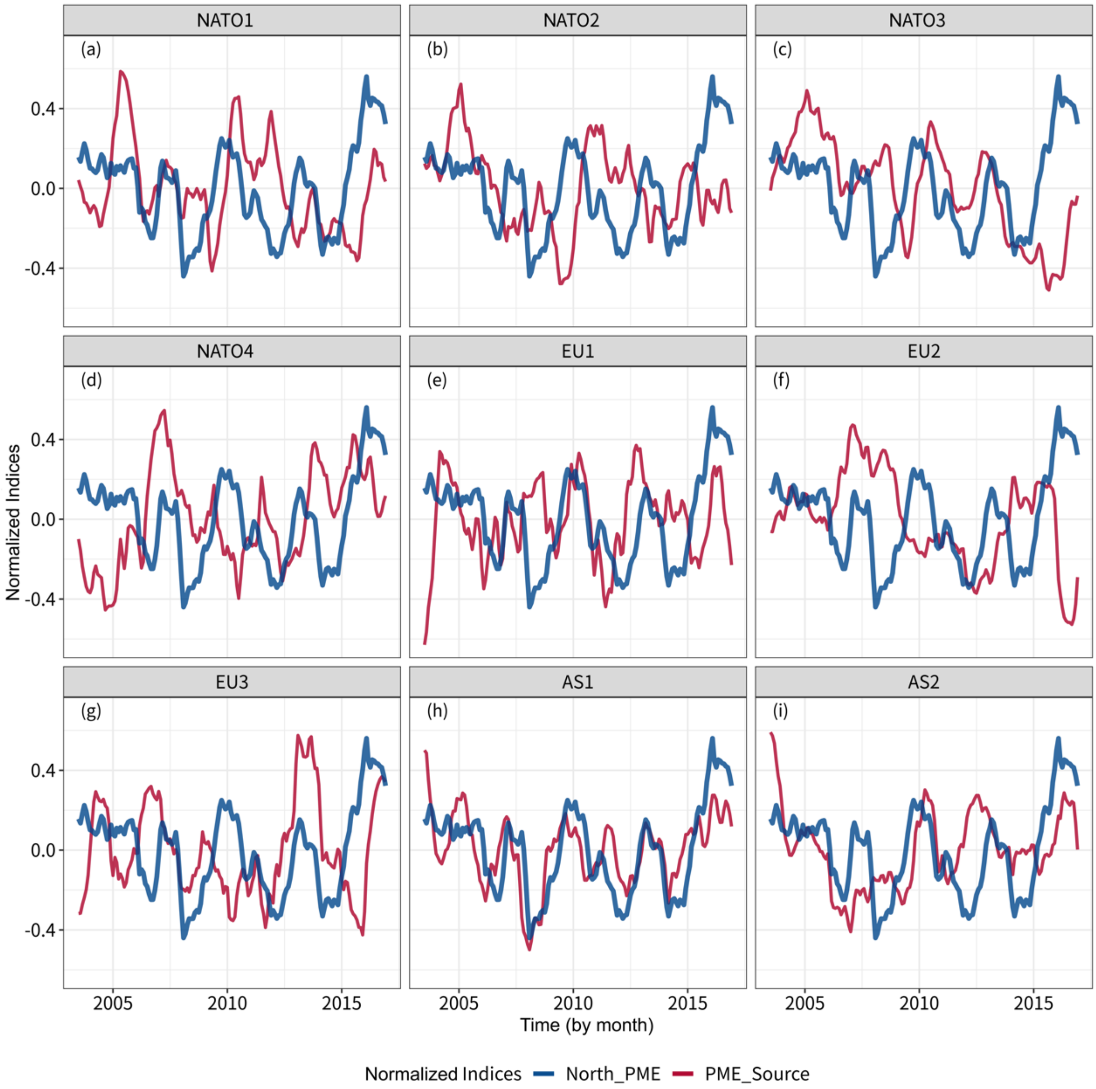

Supplementary Figure 5 Temporal variations between normalized trend items of the zonal sums of precipitation minus evapotranspiration (PME for abbreviation) in northern Xinjiang and normalized trend items of the zonal sums of PMEs in sub-regions of the northern Atlantic Ocean (NATO1-4, a-d), Europe (EU1-3, e-g), and Asia (AS1-2, h-i), respectively.

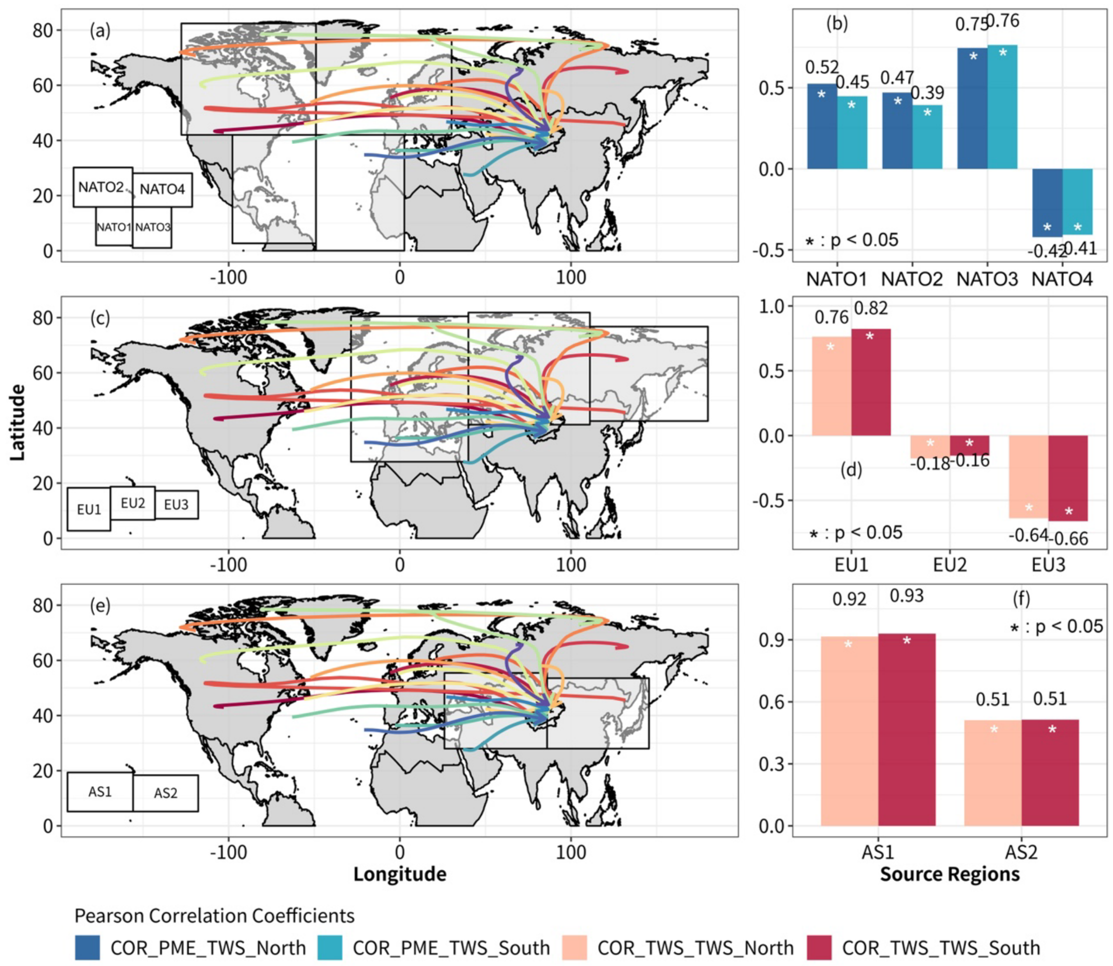

Supplementary Figure 6 Pearson correlation analysis between normalized trend items of the zonal sum of terrestrial water storage (TWS) in north (south) Xinjiang and normalized trend items of the zonal sums of precipitation-minus-evapotranspiration (PMEs) in sub-regions of the northern Atlantic Ocean (NATO1-4, a-b), normalized trend items of the zonal sums of TWSs in sub-regions of Europe (EU1-3, c-d) and Asia (AS1-2, e-f), respectively.

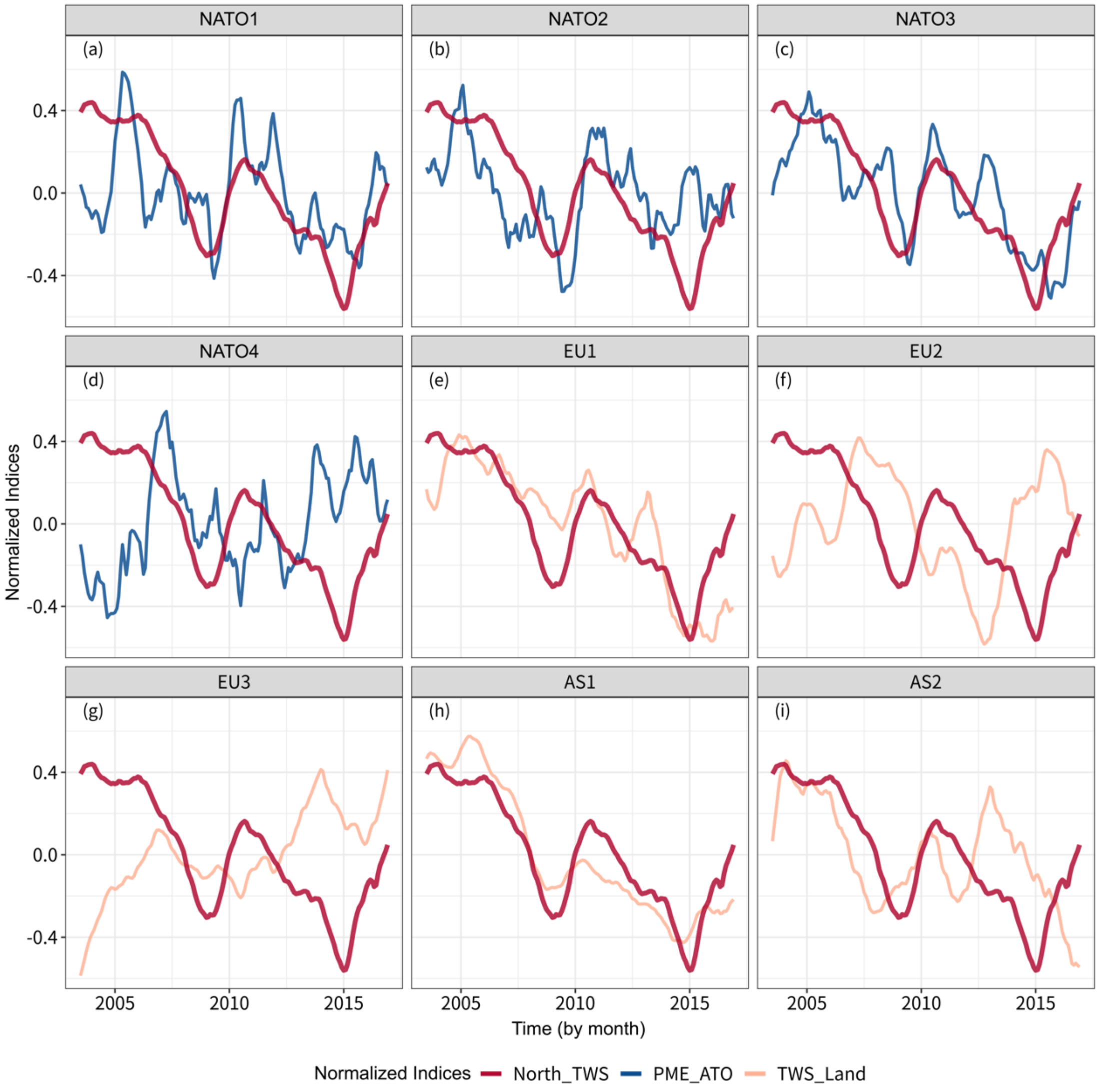

Supplementary Figure 7 Temporal variations between normalized trend items of the zonal sums of precipitation-minus-evapotranspiration (PMEs) in northern Xinjiang and normalized trend items of the zonal sums of PMEs in sub-regions of northern Atlantic Ocean (NATO1-4, a-d), normalized trend items of the zonal sums of terrestrial water storages (TWSs) in sub-regions of Europe (EU1-3, e-g) and Asia (AS1-2, h-i), respectively.

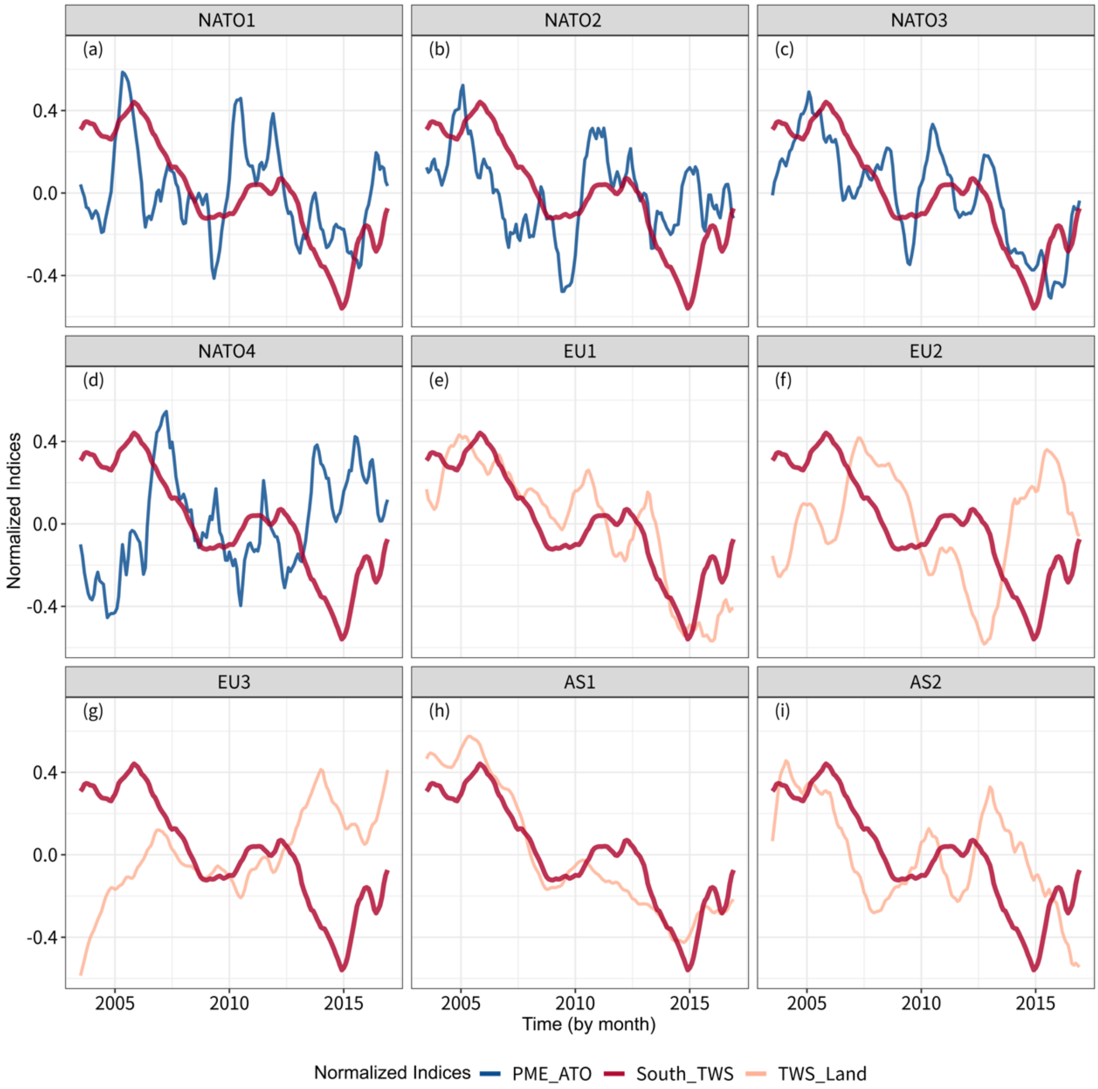

Supplementary Figure 8 Temporal variations in normalized trend items of the zonal sums of precipitation-minus-evapotranspiration (PMEs) in southern Xinjiang and normalized trend items of the zonal sums of PMEs in sub-regions of the North Atlantic Ocean (NATO1-4, a-d), normalized trend items of the zonal sums of terrestrial water storages (TWSs) in sub-regions of Europe (EU1-3, e-g) and Asia (AS1-2, h-i), respectively.

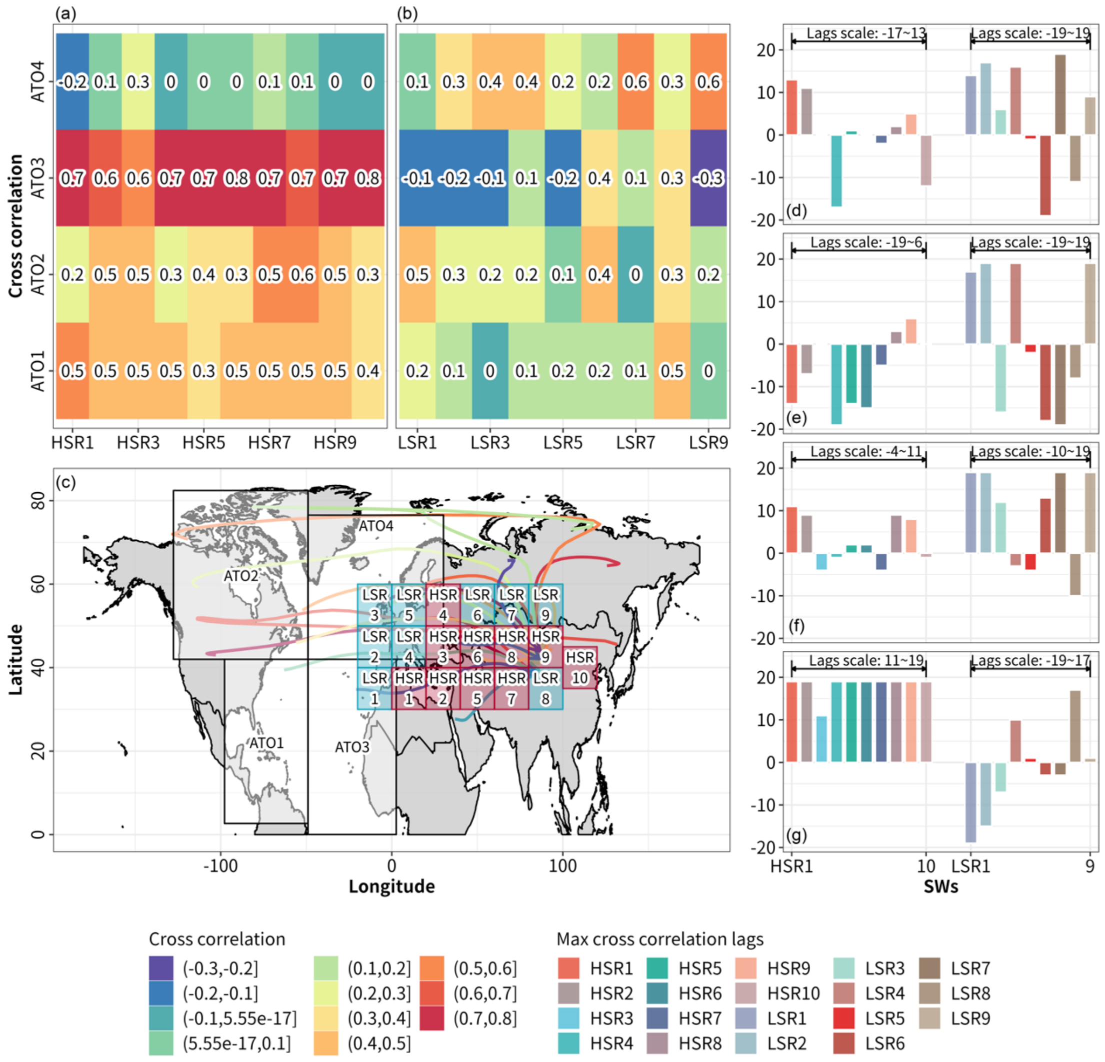

Supplementary Figure 9 Exploration in the cross correlations between normalized trend items of the zonal sums of precipitation-minus-evapotranspiration (PMEs) in NATO1-4 and normalized trend items of the zonal sums of terrestrial water storages (TWSs) in smaller sub-regions (SRs) across the mid-latitude (N30° - N60°) Eurasia along the trajectories of water vapor fluxes (a-b). We define 10 sub-regions where TWSs are highly correlated with PME in NATO3 (the cross correlation > 0.50) as high-correlated sub-regions (HSR), and define other sub-regions as low-correlated sub-regions (LSR). (c) displays the spatial distribution of NATO1-4, LSRs, and HSRs respectively. (d-g) are the lags for maximum cross correlations between TWSs in HSRs (LSRs) and PMEs in NATOs1-4, respectively.

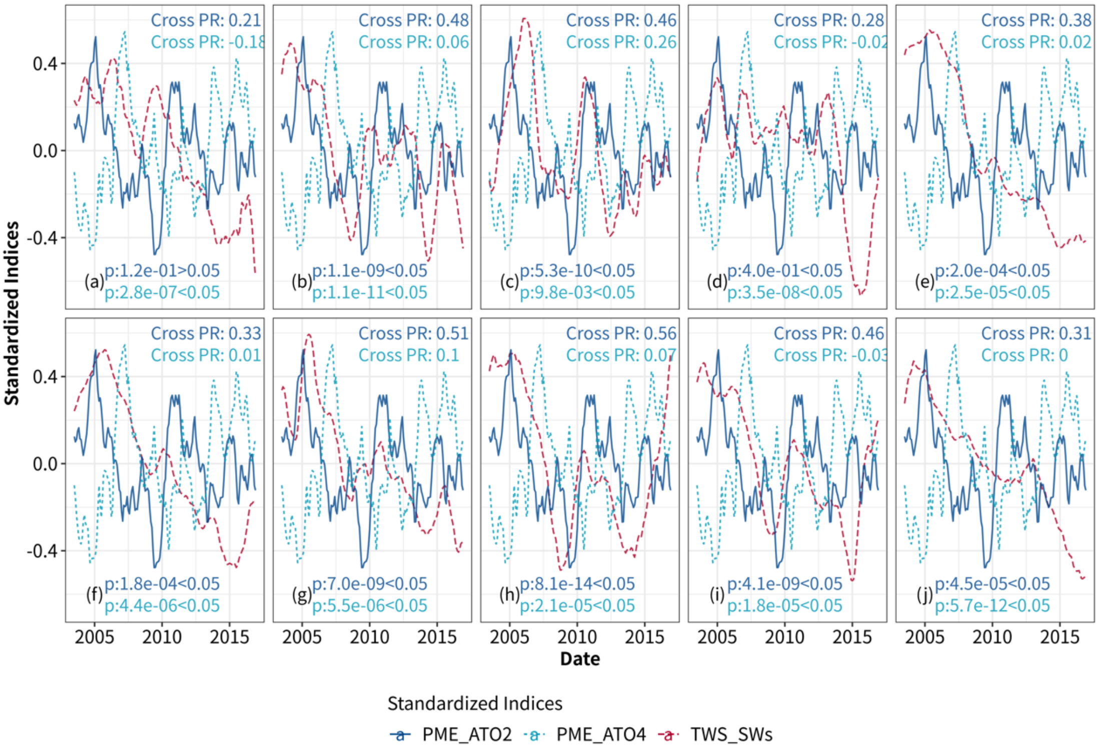

Supplementary Figure 10 Temporal variations in the normalized trend items of zonal sums of TWSs in sub-regions 1-10 (HSRs 1-10), and the normalized trend items of zonal sums of PMEs in the sub-regions 2 and 4 of North Atlantic Ocean (NATO2 and NATO4) during 2003-2017 by month (a-j). The Cross PR in the plot refers to the cross correlation coefficient.

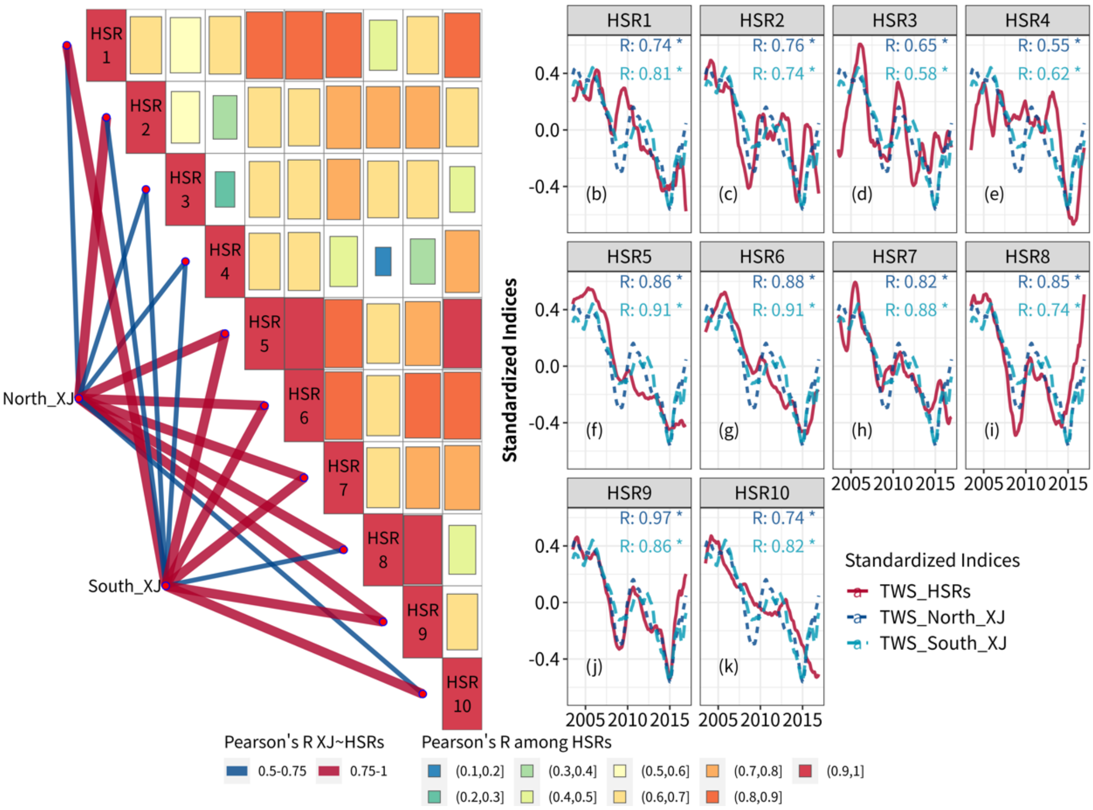

Supplementary Figure 11 Pearson correlation analysis between the normalized trend items of the regional sums of terrestrial water storages (TWSs) in the north (south) Xinjiang and normalized trend items of the regional sums of TWSs in HSR1-10 (a). (b-k) are the temporal variations in the normalized TWSs in north (south) Xinjiang and normalized TWSs in HSRs 1-10. The blue * marks the p.value < 0.05.

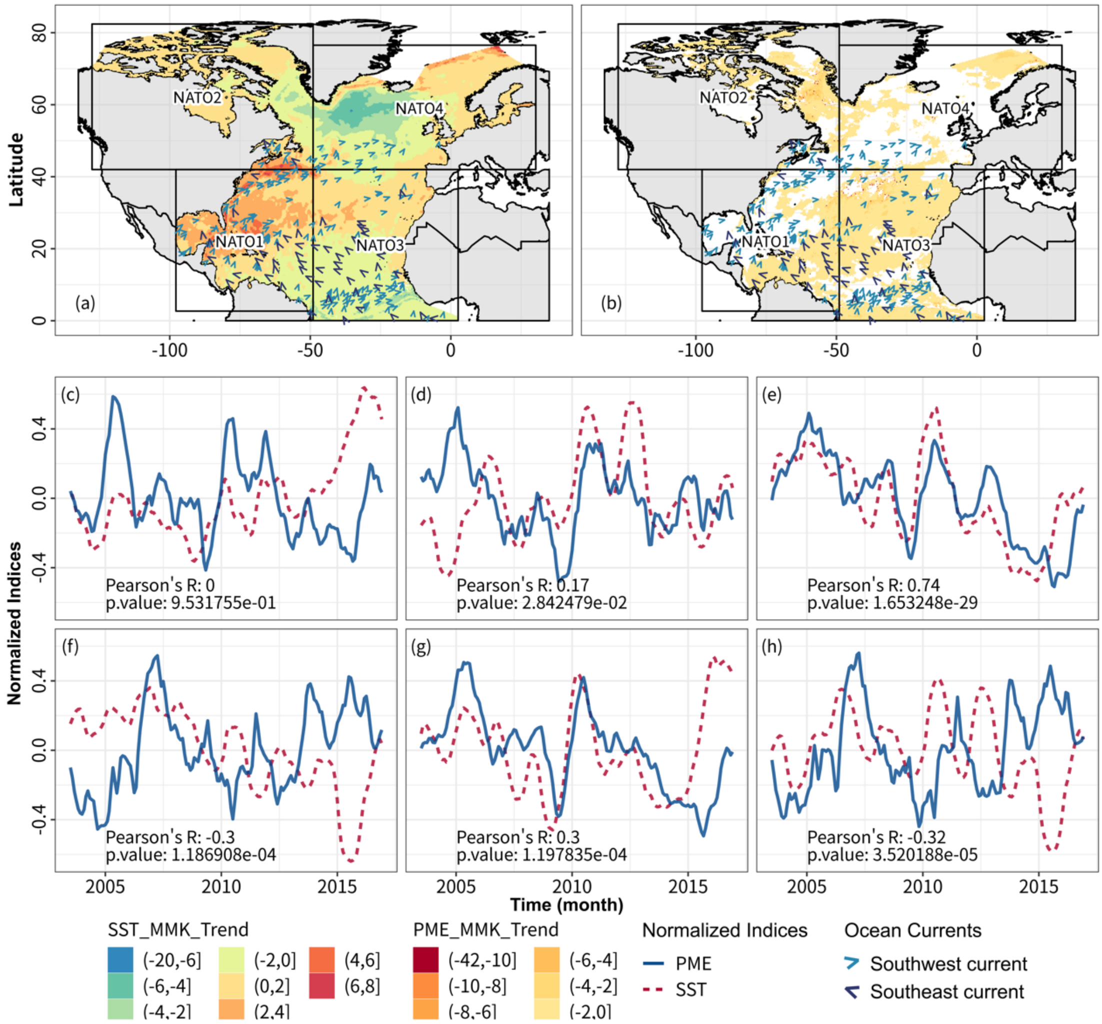

Supplementary Figure 12 Spatial patterns of the modified Mann-Kendall (MMK) trends of surface sea temperature (SST, a) and precipitation-minus-evapotranspiration (PME, b) in the northern Atlantic Ocean (NATO). Temporal covariations between normalized trend items of regional averaged SST in NATO1-4 and normalized trend items of regional sums of PMEs in NATO1-4 (c-f), the normalized trend item of regional sums of PMEs in NATO1 and NATO3(g), the normalized trend item of regional sums of PMEs in NATO2 and NATO4(h), respectively.

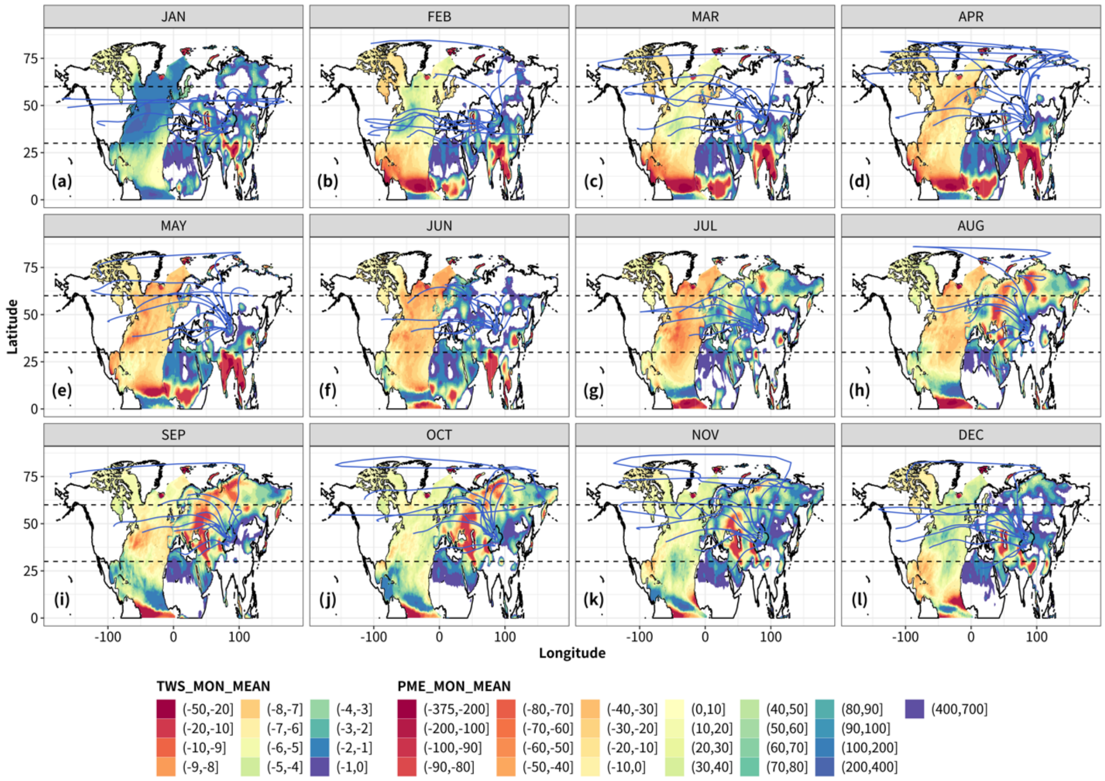

Supplementary Figure 13 Spatial patterns of the monthly mean precipitation-minus-evapotranspiration (PME) anomalies in the North Atlantic Ocean and the monthly mean terrestrial water storage (TWS) anomalies across Europe, Asia and North Africa from January to December during 2003-2017 (a-l). The blue trajectories in the plot refer to monthly mean clustered moisture trajectories.

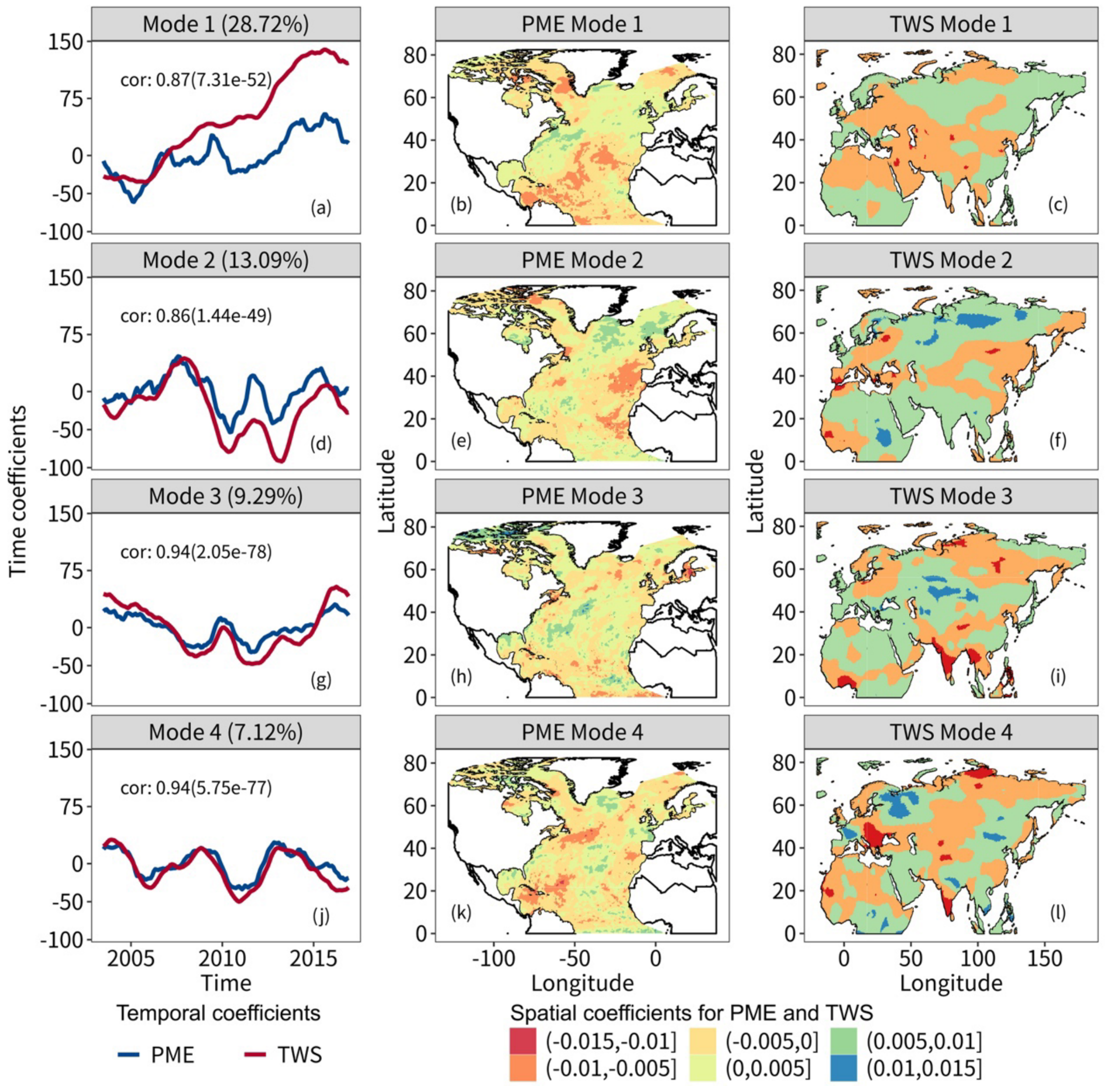

Supplementary Figure 14. Maximum covariance analysis (MCA) between TWS across Eurasia and PME over NATO. (a,d,g,j) are the temporal sequences for TWS and PME in the leading modes 1-4. (b,e,h,k) are the spatial modes of PME in the leading modes 1-4. (c,f,i,l) are the spatial modes of TWS in the leading modes 1-4.

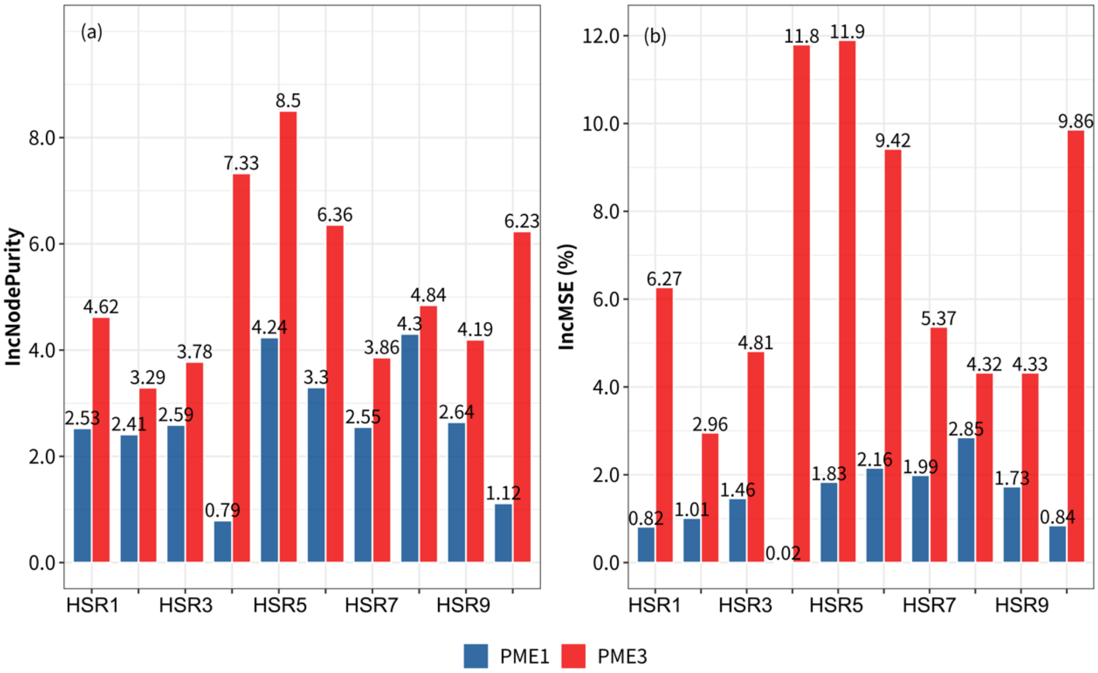

Supplementary Figure 15 Importance matrixes of the random forest regression models for the projections of the terrestrial water storage in HSRs 1-10 based on the variations in the precipitation-minus-evapotranspiration (PMEs) in both NATO1 and NATO3. And the importance matrixes contain IncNodePurity (a) and IncMSE(%) (b). Two indices all demonstrated that the variation in PME over NATO3 dominated the variations in TWSs in HSR1-10 across Eurasia in comparison with the variation in PME over NATO1.

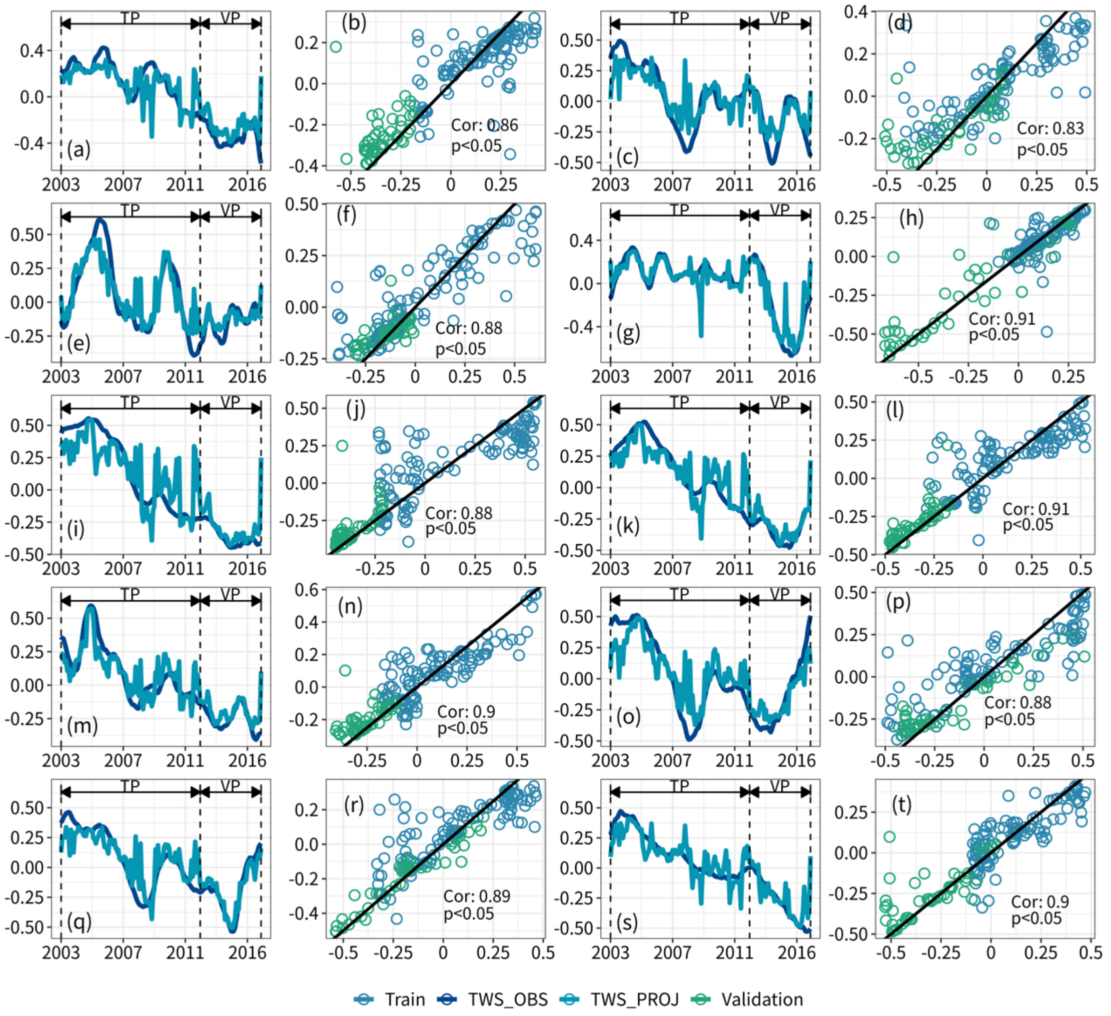

Supplementary Figure 16 In-order training (2003-2012) and validation (2012-2016) period of the random forest regression models for the projections of TWS in HSRs 1-10 based on the variations in the precipitation-minus-evapotranspiration (PMEs) in NATO1 and NATO3. (a,c,e,g,i,k,m,o,q,s) show the temporal covariations between projected and observed TWSs in HSRs 1-10, respectively. And (b,d,f,h,j,l,n,p,r,t) present the comparisons between projected and observed TWSs during training and validation periods in HSRs 1-10, respectively. Note that, the random forest regression models were firstly trained and validated based on the 70/30 randomly separated data, and the best models were selected to be trained and validated based on the 70/30 in-order data (2003-2012 and 2012-2016). The whole study period is from January 2003 to June 2017, since there are 12 NA values in the process of extraction of trend items, the period for trend items is from July 2003 to December 2016.

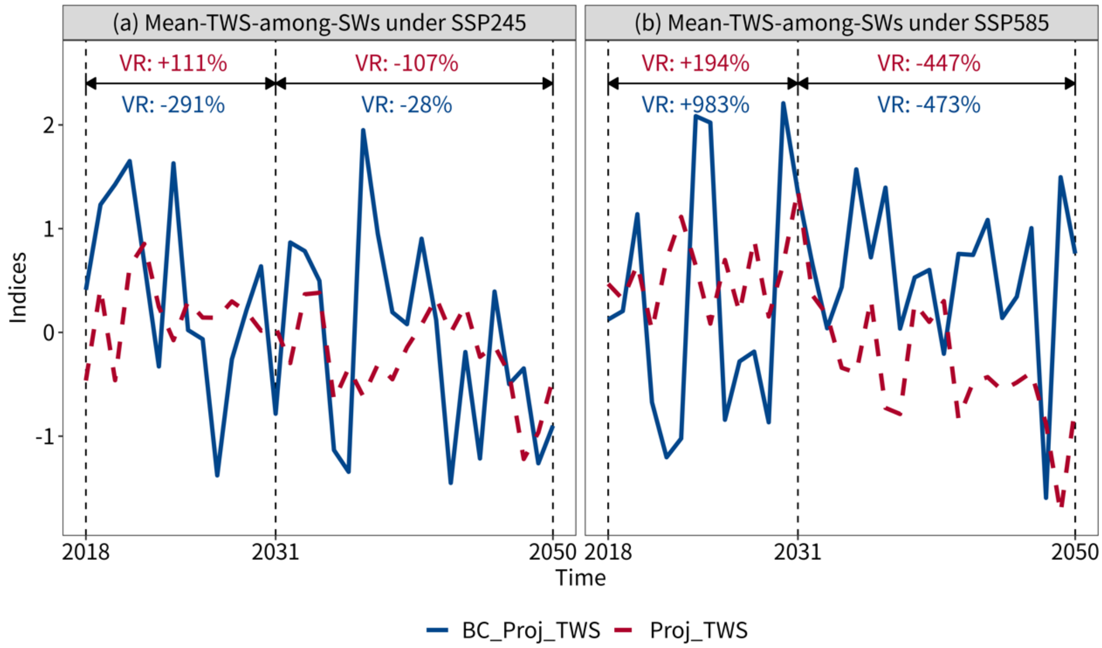

Supplementary Figure 17. Comparisons between the projected TWS across the mid-latitude Eurasia based on the projected PME by the original simulation of CMIP6 models (Proj_TWS) and weighted CMIP6 models ensemble (BC_Proj_TWS) over NATO1 and NATO3 under SSP245 scenario (a) and SSP585 scenario (b) during 2018-2050.

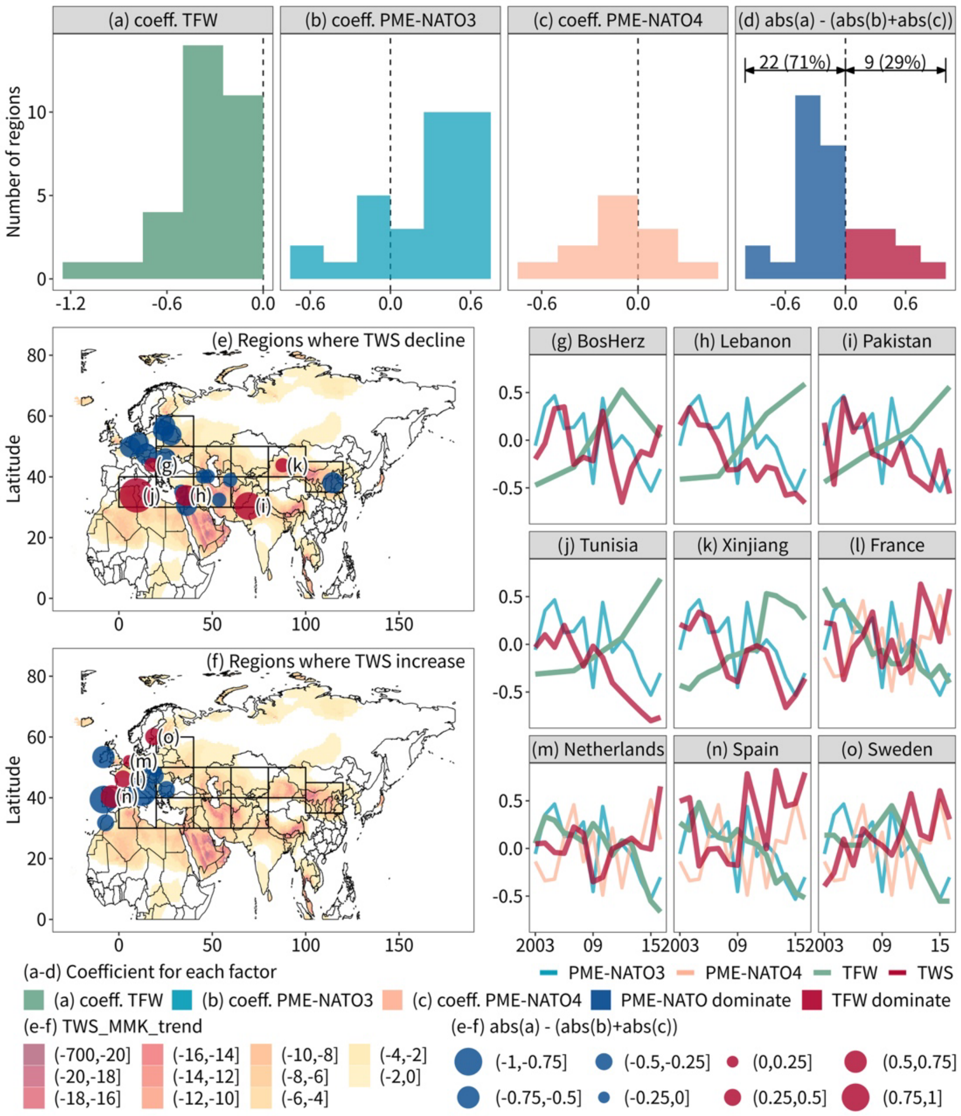

Supplementary Figure 18 Attribution analysis for the variation in the TWS across the Eurasia on the region/country scale. (a-c) refer to the coefficients for the TFW, PME over the NATO3 and PME over the NATO4, respectively. (d) refers to the difference between the absolute value of the coefficient a and sum of the absolute value of the coefficient b and c. And the negative difference value indicates the variation in TWS is majorly influenced by the TFW (TFW-dominated TWS) while the positive difference value indicates the variation in TWS is majorly influence by the variation in PME over the NATO (PME-NATO-dominated TWS). (e-f) refer to the spatial patterns of the regions with the TFW-dominated TWS or PME-NATO-dominated TWS where the TWS were in declining (e) or increasing (f) trends. (g-o) refer to the temporal variations in TWS, TFW, PME-NATO3 and PME-NATO4 at regions with TFW-dominated TWS during 2003-2016.

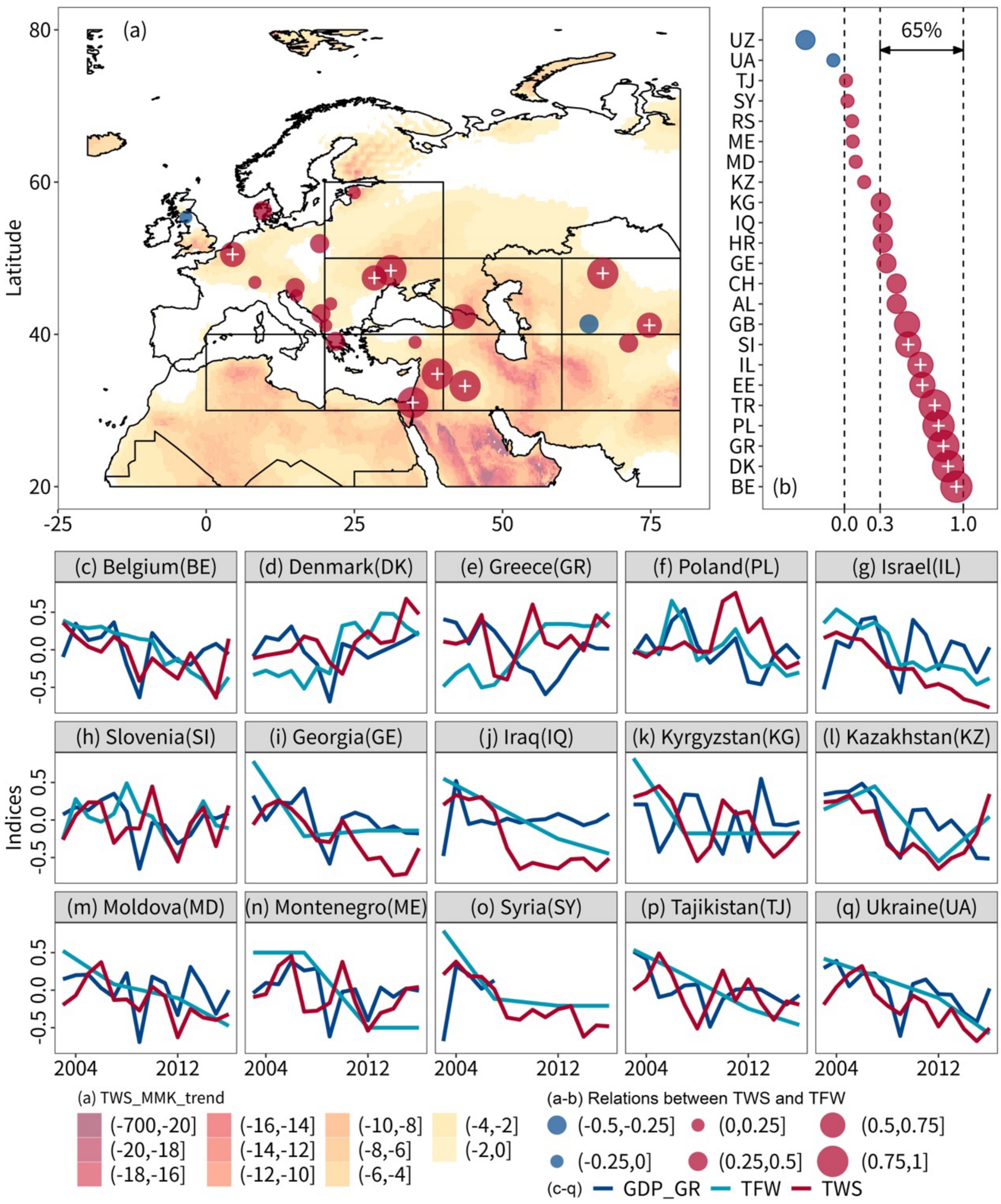

Supplementary Figure 19. The impact induced by the decline in TWS across the mid-latitude Europe on the consume behavior of the freshwater resource and local economy development. (a) Spatial pattern of the modified Mann-Kendall trends of the TWS and relations between TWS and TFW on the country/region scale. (b) Statistic analysis of the relations between TWS and TFW on the country/region scale. The white cross marks the significant relation with the p.value < 0.05. (c-q) Temporal variations in the TWS, TFW and the growth rate of the Gross Domestic Product (GDP_GR).

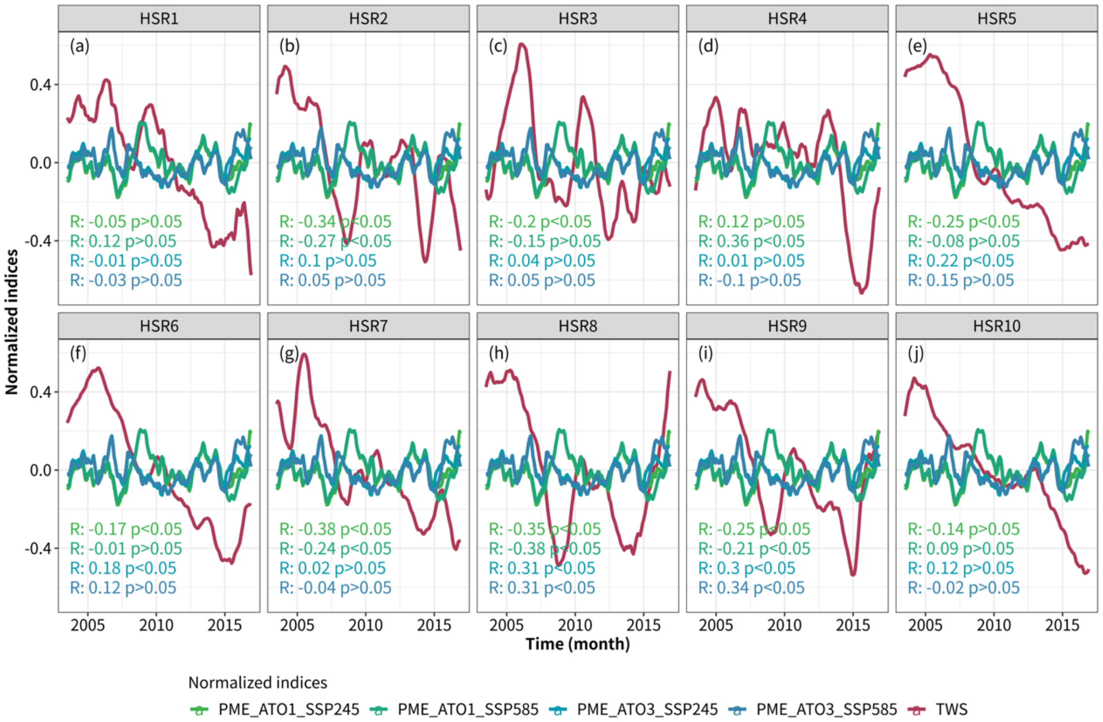

Supplementary Figure 20 Temporal covariations between PMEs in NATO1 and NATO3 and TWSs in HSR1-10 (a-j) during 2003-2016, respectively. In the plot, R refers to the Pearson correlation coefficient and p refers to the significant level.

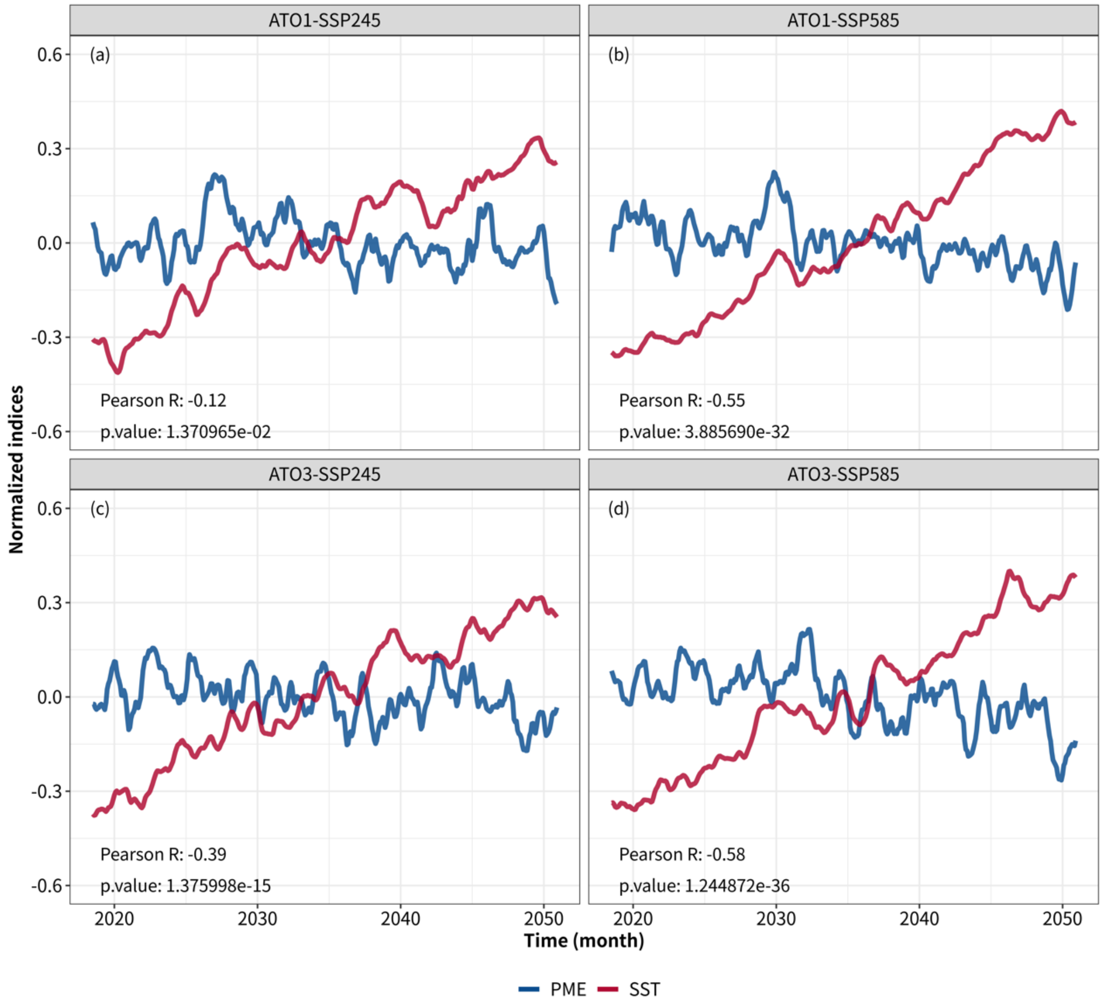

Supplementary Figure 21 Temporal covariations between the normalized trend items of the regional sums of precipitation-minus-evapotranspiration (PMEs) and the normalized trend items of the regional means of surface sea temperature (SSTs) in the NATO1 under SSP245 (a) and SSP585 (b), and in the NATO3 under SSP245 (c) and SSP585 (d) during 2018-2050 by month, respectively.

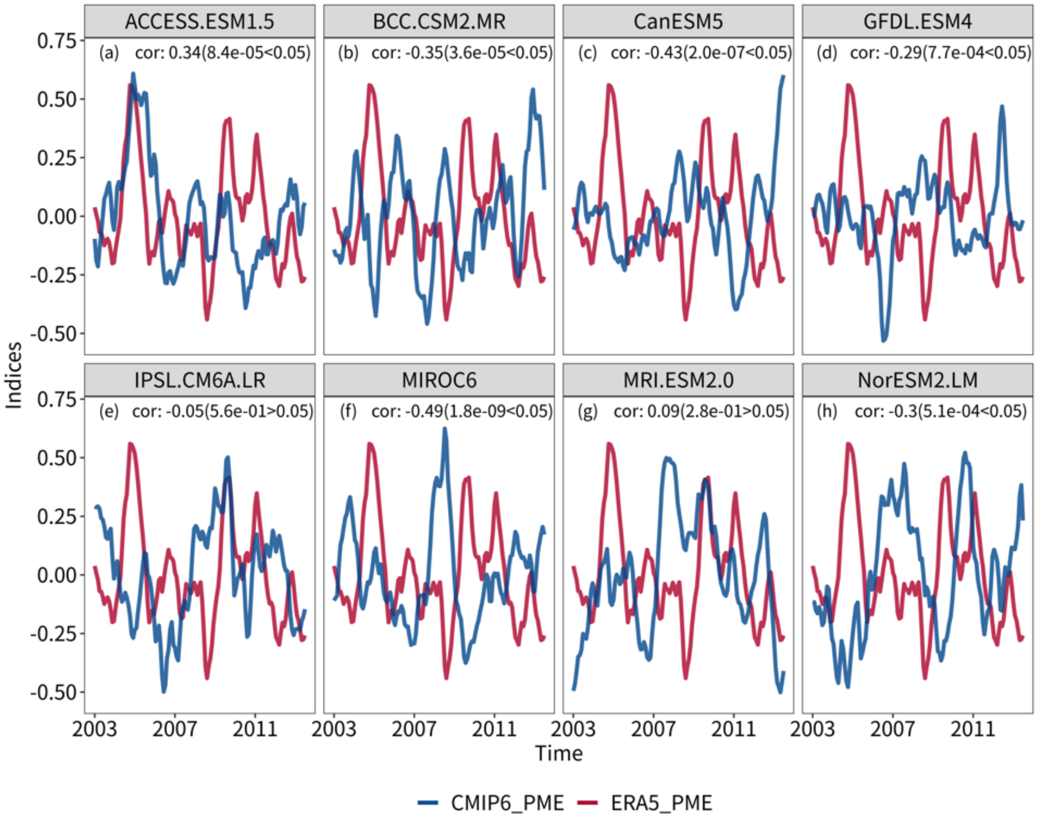

Supplementary Figure 22. Evaluation of the CMIP6-PME over the NATO1 in comparison with the ERA5-PME during 2003-2014. And (a-h) refers to comparison between ERA5-PME over NATO1 and CMIP6-PMEs from ACCESS-ESM1-5 (a), BCC-CSM2-MR (b), CanESM5 (c), GFDL-ESM4 (d), IPSL-CM6A-LR (e), MIROC6 (f), MRI-ESM2-0 (g) and NorESM2-LM (h), respectively.

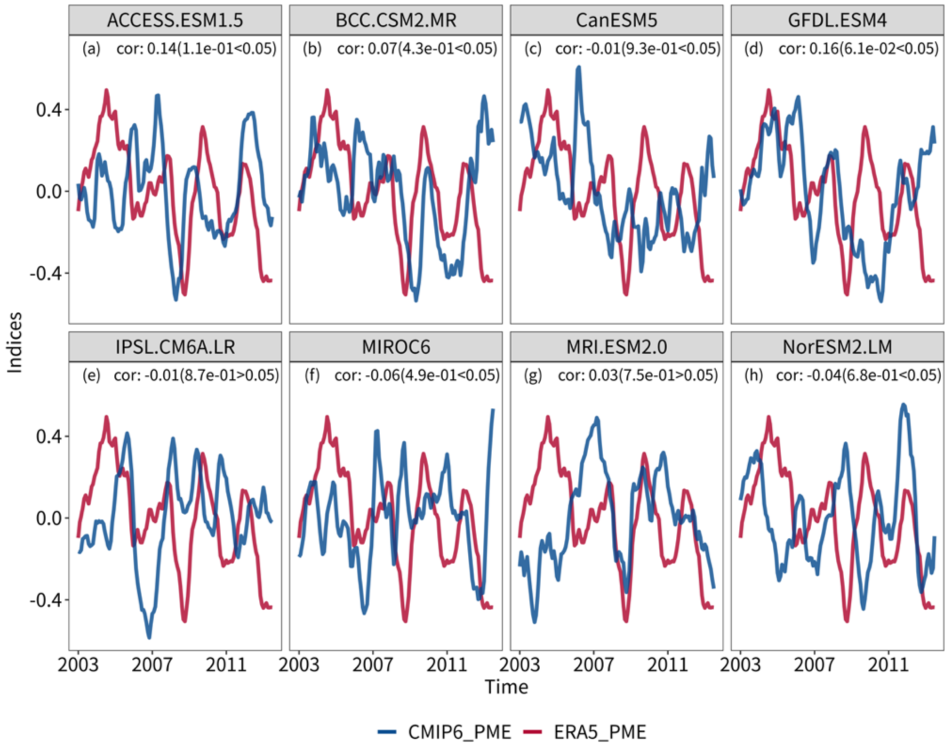

Supplementary Figure 23. Evaluation of the CMIP6-PME over the NATO3 in comparison with the ERA5-PME during 2003-2014. And (a-h) refers to comparison between ERA5-PME over NATO3 and CMIP6-PMEs from ACCESS-ESM1-5 (a), BCC-CSM2-MR (b), CanESM5 (c), GFDL-ESM4 (d), IPSL-CM6A-LR (e), MIROC6 (f), MRI-ESM2-0 (g) and NorESM2-LM (h), respectively.

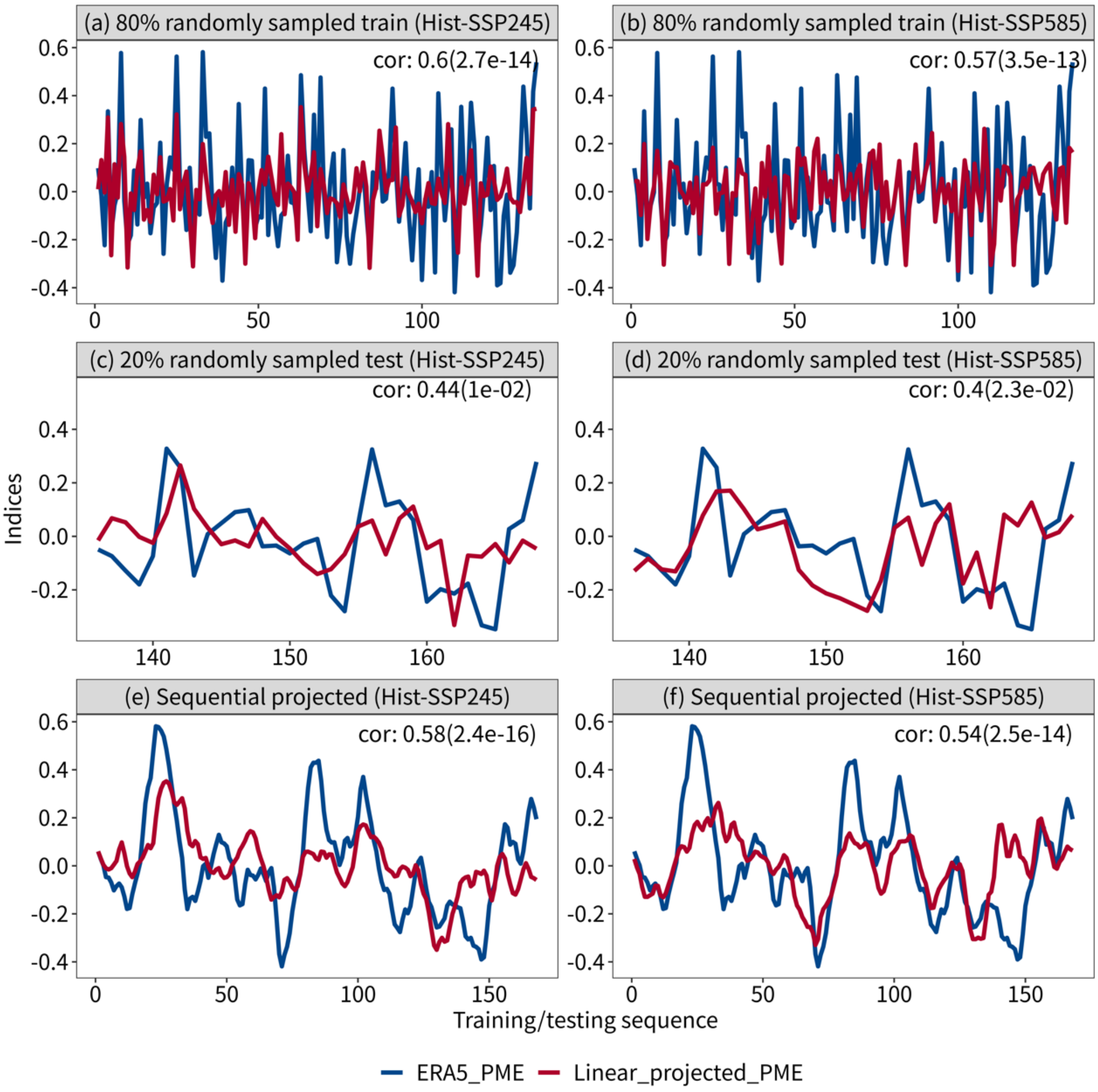

Supplementary Figure 24. Evaluation of the simulated PMEs by the weighted CMIP6 models ensemble over the NATO1 under historical-SSP245 and historical-SSP585 scenarios. (a-b) refer to the temporal variations in ERA5-PME and simulated PMEs based on the 80% of the randomly sampled training dataset under historical-SSP245 and historical-SSP585 scenarios. (c-d) refer to the temporal variations in ERA5-PME and simulated PMEs based on the 20% of the randomly sampled testing dataset under historical-SSP245 and historical-SSP585 scenarios. (e-f) refer to the temporal variations in ERA5-PME and simulated PMEs by the weighted CMIP6 models ensemble during 2003-2017.

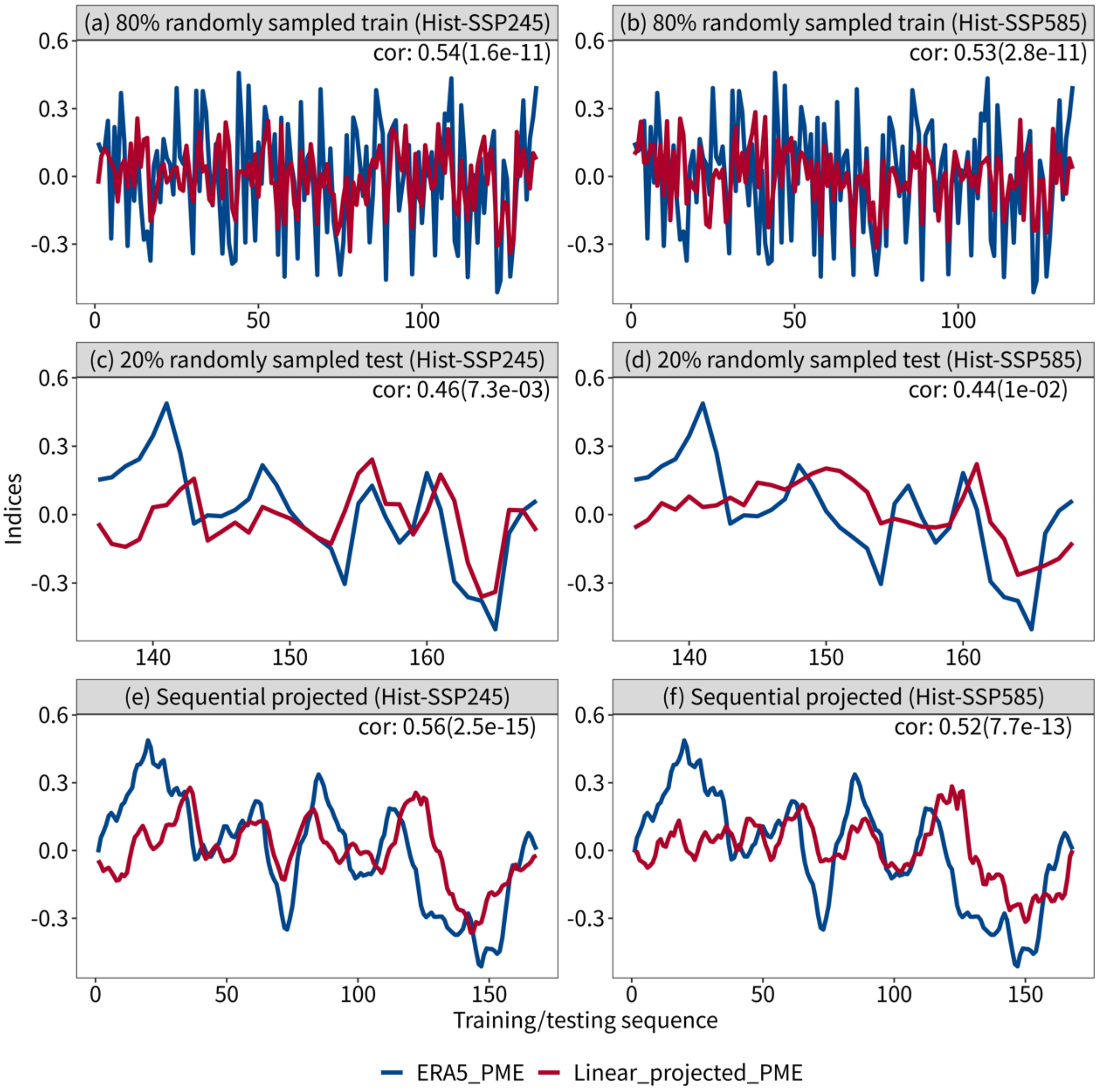

Supplementary Figure 25. Evaluation of the simulated PMEs by the weighted CMIP6 models ensemble over the NATO3 under historical-SSP245 and historical-SSP585 scenarios. (a-b) refer to the temporal variations in ERA5-PME and simulated PMEs based on the 80% of the randomly sampled training dataset under historical-SSP245 and historical-SSP585 scenarios. (c-d) refer to the temporal variations in ERA5-PME and simulated PMEs based on the 20% of the randomly sampled testing dataset under historical-SSP245 and historical-SSP585 scenarios. (e-f) refer to the temporal variations in ERA5-PME and simulated PMEs by the weighted CMIP6 models ensemble during 2003-2017.

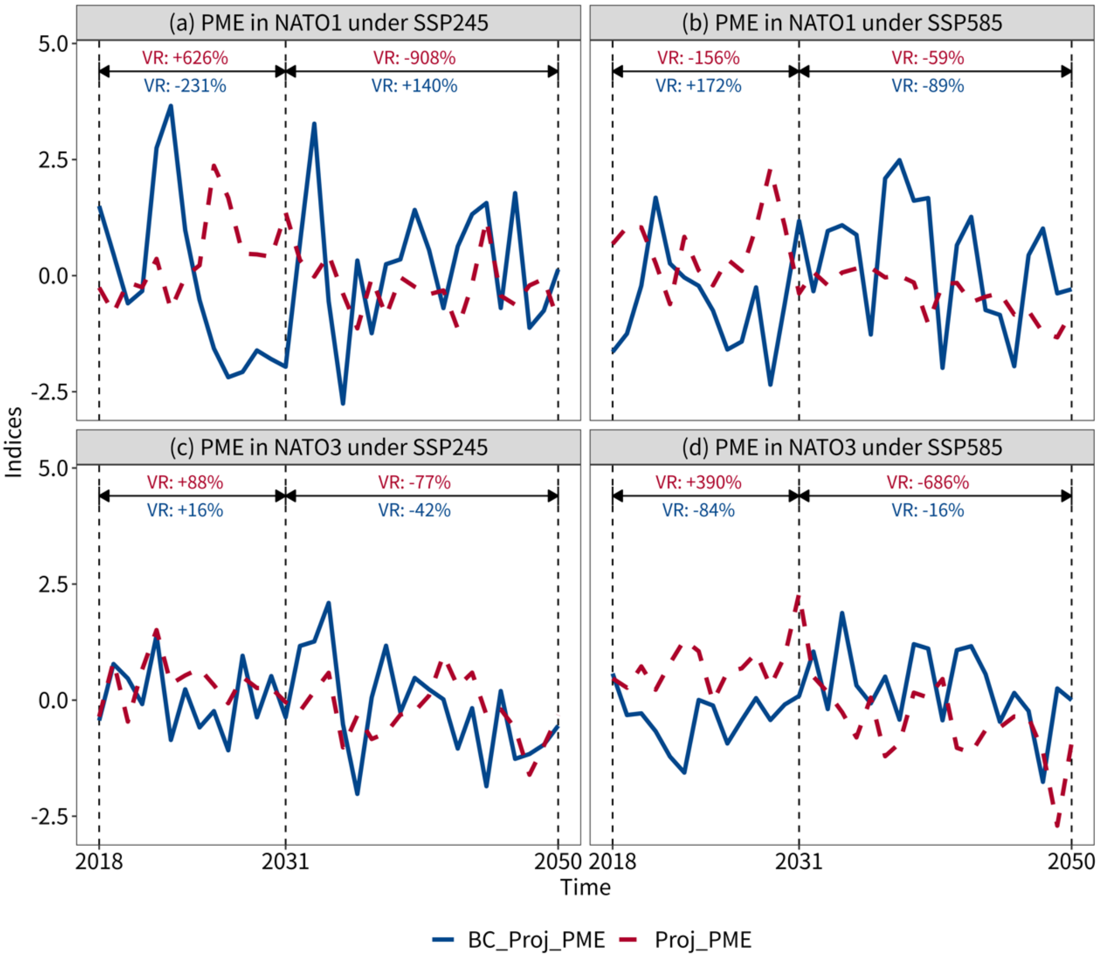

Supplementary Figure 26. Comparisons between the projected PME based on the original simulation of the CMIP6 models (Proj_PME) and weighted CMIP6 models ensemble (BC_Proj_PME) over the NATO1 under SSP245 (a) and SSP585 (b) scenarios during 2018-2050. Comparisons between the projected PME based on the Proj_PME and BC_Proj_PME over the NATO3 under SSP245 (c) and SSP585 (d) scenarios during 2018-2050.

Supplementary Table 1. A summary of the abbreviation use in the main text

Index	Abbreviation	Clarification
1	MLE	mid-latitude Eurasia
2	TWS	Terrestrial water storage
3	LNATO	low-latitude North Atlantic Ocean
4	PME	precipitation-minus-evapotranspiration
5	GRACE	Gravity Recovery and Climate Experiment
6	ENSO	El Niño-Southern Oscillation
7	NATO	North Atlantic Ocean
8	NATO1-4	sub-regions 1-4 of NATO
9	ERA5	ECMWF Reanalysis 5th generation
10	CMIP6	Coupled Model Intercomparison Project Phase 6
11	SSP245	Shared Socioeconomic Pathway 2-4.5
12	SSP585	Shared Socioeconomic Pathway 5-8.5
13	FLEXPART	Lagrangian transport and dispersion model
14	XJ	Xinjiang
15	AS	AS, Asia excluding Xinjiang
16	EU	Europe including Russia
17	TCR	total contribution rate (TCR)
18	GPCC	Global Precipitation Climatology Centre
19	CR	Contribution rates
20	EU1-3	sub-regions 1-3 of EU
21	AS1-2	sub-regions 1-2 of EU
22	NATO1-4	sub-regions 1-4 of NATO
23	HSR	sub-regions where TWSs were highly correlated with PME in NATO3 (the cross correlation $>$ 0.50) as high-correlated sub-regions
24	LSR	sub-regions where TWSs were lowly correlated with PME in NATO3 (the cross correlation $<$ 0.50 as low-correlated sub-regions (LSR)
25	MMK	Modified Mann-Kendall trend
26	SST	Sea Surface Temperature
27	NCP	Northern China Plain
28	CS	Caspian Sea
29	NAF	North Africa
30	NAM or NA	North America
31	MS	Mediterranean Sea
32	AO	Arctic Ocean
33	BS	Black Sea
34	IO	Indian Ocean
35	RS	Red Sea
36	PO	Pacific Ocean
37	TFW	Total Freshwater Withdrawal

Supplementary Table 2. The normalized trend item of every index in the study

ID	Full names	Abbreviation	Calculation	Data source
1	Normalized trend item of regional sum of the precipitation minus evapotranspiration (PME)	PME	$\frac{PME - mean\ (PME)}{max(PME) - min\ (PME)}$	ERA5 and GPCC, respectively
2	Normalized trend item of regional sum of the terrestrial water storage (TWS)	TWS	$\frac{TWS_{anomaly}}{max\left(TWS_{anomaly}\right) - min\ (TWS_{anomaly})}$	GRACE
3	Normalized trend item of regional mean of the sea surface temperature (SST)	SST	$\frac{SST - mean\ (SST)}{max(SST) - min\ (SST)}$	ERA5
4	Normalized trend item of total contribution rates (TCR)	TCR	$\frac{TCR - mean\ (TCR)}{max(TCR) - min\ (TCR)}$	Outputs of the FLEXPART driven by ERA-interim

Note: In this study, all indices abbreviation referred to the normalized trend items of the regional sums of the indices if there is no specific denotation. Since the GRACE TWS data was the anomaly of the TWS, the normalization of TWS was different from others.
